# Gait Characteristics under Imposed Challenge Speed Conditions in Patients with Parkinson’s Disease During Overground Walking

**DOI:** 10.3390/s20072132

**Published:** 2020-04-10

**Authors:** Myeounggon Lee, Changhong Youm, Byungjoo Noh, Hwayoung Park, Sang-Myung Cheon

**Affiliations:** 1 Biomechanics Laboratory, College of Health Sciences, Dong-A University, Hadan 2-dong, Saha-gu, Busan 49315, Korea; ssam011@dau.ac.kr (M.L.); app00113@dau.ac.kr (H.P.); 2 Department of Healthcare and Science, College of Health Sciences, Dong-A University, 37 Nakdong-Daero, 550 Beon-gil, Hadan 2-dong, Saha-gu, Busan 49315, Korea; bnoh@dau.ac.kr; 3 Department of Neurology, School of Medicine, Dong-A University, Dongdaesin-dong 3-ga, Seo-gu, Busan 49315, Korea; smcheon@dau.ac.kr

**Keywords:** Parkinson’s disease, inertial measurement unit (IMU), walking speeds, gait variability, risk of falling

## Abstract

Evaluating gait stability at slower or faster speeds and self-preferred speeds based on continuous steps may assist in determining the severity of motor symptoms in Parkinson’s disease (PD) patients. This study aimed to investigate the gait ability at imposed speed conditions in PD patients during overground walking. Overall, 74 PD patients and 52 age-matched healthy controls were recruited. Levodopa was administered to patients in the PD group, and all participants completed imposed slower, preferred, and faster speed walking tests along a straight 15-m walkway wearing shoe-type inertial measurement units. Reliability of the slower and faster conditions between the estimated and measured speeds indicated excellent agreement for PD patients and controls. PD patients demonstrated higher gait asymmetry (GA) and coefficient of variance (CV) for stride length and stance phase than the controls at slower speeds and higher CVs for phases for single support, double support, and stance. CV of the double support phase could distinguish between PD patients and controls at faster speeds. The GA and CVs of stride length and phase-related variables were associated with motor symptoms in PD patients. Speed conditions should be considered during gait analysis. Gait variability could evaluate the severity of motor symptoms in PD patients.

## 1. Introduction

Parkinson’s disease (PD) is a progressive neurodegenerative disorder characterized by poor coordination and sustained gait impairments, such as decreased step length, stride length, and walking speed, which may increase the risk of falling [1,2,3,4]. Increasing the walking speed may increase the risk of falling in PD patients [2,3,5]. PD patients present with sustained motor symptoms, which may adversely affect their stability during a faster walking task [2]. Slower or faster speed conditions, which commonly arise in daily life, may increase the risk of injuries and falls; thus, assessment of walking speed may be useful for predicting the risk of falling in PD patients [3].

Previous studies analyzed gait at slower or faster speeds in PD patients [2,6,7,8] and healthy participants [9,10,11,12]. However, walking speeds were not controlled using quantitative values [6,7,9,12], which may have resulted in higher deviations in speed conditions. Other studies have controlled walking speeds using quantitative values [2,8,10,11], but this may have led to an exaggeration of the results because the speeds used were too slow or too fast (speeds were 10% to 50% different from self-preferred speeds). Furthermore, most of the previous studies used averaged data from repeated trials to collect multiple steps, which may not accurately replicate the natural walking patterns of individuals [13]. Consequently, it remains unclear which speed conditions reflect a decline in gait ability in PD patients. Evaluating gait stability at slower or faster speeds and self-preferred speeds may be useful for determining the severity of motor symptoms in PD patients.

The purpose of this study was to investigate the gait ability at imposed speed conditions (slower, preferred, and faster speeds) in PD patients during overground walking. We hypothesized that PD patients and age-matched controls would exhibit excellent agreement for the estimated slower (80% of preferred speed) and faster (120% of preferred speed) speeds and for measured speeds (real measured values in the slower and faster speed tasks). We also hypothesized that PD patients would exhibit more reduced gait stability, indicated by the higher coefficient of variance (CV) for spatiotemporal parameters at the slower or faster speed conditions. 

## 2. Materials and Methods

### 2.1. Participants

We contacted 162 individuals, and 150 (85 PD patients, 65 age-matched controls) agreed to participate in this study. PD patients were identified from a medical center’s outpatient clinic, and had met the UK Brain Bank criteria for PD diagnosis [14]. Controls were age-matched, community-dwelling adults.

**Figure 1:**
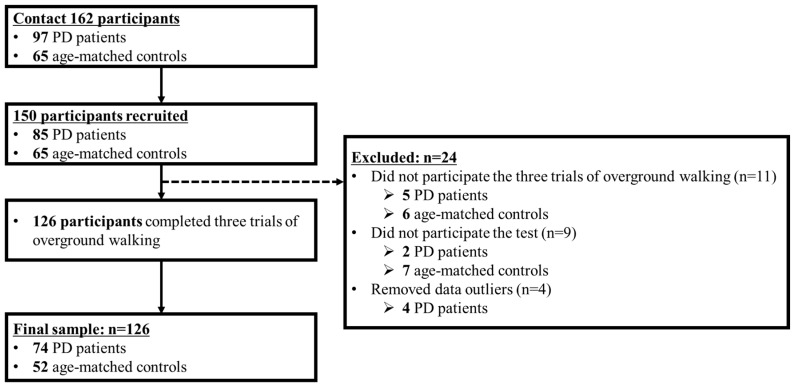
Recruitment process flowchart.

Twenty-four participants were excluded; 11 did not complete the three trials of overground walking; nine did not participate in the test; and four were data outliers. In total, 74 PD patients and 52 age-matched controls completed three trials of overground walking under slower, preferred, and faster speed conditions (Figure 1).

The PD patient group inclusion criteria were: (a) idiopathic PD diagnosis, (b) Hoehn & Yahr (H&Y) stages 1 to 3, and (c) the use of anti-Parkinson’s medication. None of the participants had any history of orthopedic, neurosurgical, or neurological problems in the preceding six months, and their Mini-Mental State Exam (MMSE) scores were more than 24 points (Table 1). All participants read and signed an informed consent form approved by the Dong-A University Institutional Review Board (IRB number: HR-025-04 [See Supplementary File S1]). The study protocol was approved by the Ethics Committee, and was performed in accordance with the guidelines of the Declaration of Helsinki of 1975, as revised in 2013.

### 2.2. Instrumentation

**Figure 2:**
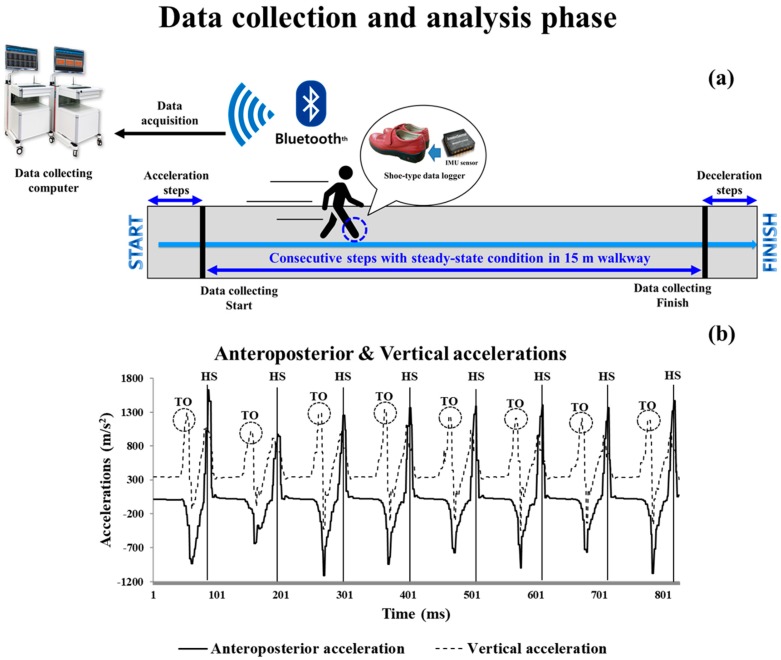
Schematic of the data collection and analysis phase under steady-state conditions. (**a**) Data collection and analysis phase; the blue arrows indicate acceleration to steady-state and deceleration steps after measurements are completed. (**b**) Detection of gait events with the shoe-type inertial measurement unit (IMU) system. Data is collected at 100 Hz. HS, heel strike; TO, toe-off.

Shoe-type inertial measurement unit (IMU) sensor-based gait analysis systems (DynaStab^TM^, JEIOS, Busan, Korea), including a shoe-type data logger (Smart Balance^®^ SB-1, JEIOS, Busan, Korea) and a data acquisition system were utilized. The shoe-type data logger included an IMU sensor (IMU–3000^TM^, InvenSense, CA, USA) that measured triaxial acceleration (up to ±6 g) and triaxial angular velocities (up to ±500° s^−1^) along the three orthogonal axes [15,16]. The IMU sensors, available in multiple sizes to fit the shoe sizes of all participants, were installed on the outsoles of both shoes. The data were transmitted wirelessly to a data acquisition system via Bluetooth^®^ (Figure 2). The spatiotemporal variable of the shoe-type IMU system indicated excellent data agreement compared to that of a three-dimensional motion capture system for PD patients (intraclass correlation coefficient (ICC) range: 0.944–0.985) [16].

### 2.3. Test Procedures

Before the overground walking test, all PD patients were assessed by a PD specialist using the unified Parkinson’s disease rating scale (UPDRS), H&Y stage, MMSE, and disease duration. All PD patients took medications to treat Parkinson’s disease at least three hours before the tests (ON-state), because this replicates the condition in which most PD patients perform daily activities [17]. A detailed list of Parkinson’s medications is shown in Supplementary Table S1.

All participants completed three trials of the overground walking test along a straight 15-m walkway at slower, preferred, and faster speeds wearing shoe-type embedded IMU sensors. The preferred speed was the participant’s comfortable and stable walking speed without the requirement of any support during overground walking, calculated as distance (15-m) by elapsed time. The slower and faster speeds were calculated relative to the preferred speed, i.e., 20% slower or faster than the preferred speed [10,18]. All walking speeds were defined using a metronome (beats/min). The participants were asked to walk at the preferred speed to measure cadence using a metronome before each trial. An experimental operator explained the estimated walking speeds to participants before each trial. Participants were instructed to perform the overground walking test at speeds as close as possible to the targeted walking speeds, as described previously [19]. Although real stepping rhythms and measured metronome beats may not have demonstrated perfect agreement, we attempted to control the accuracy of each participants’ performance as much as possible. The metronome was turned off when the participants were engaged in the test trials because an external auditory cue during walking might enhance the performance of PD patients [8,20]. All participants practiced all speed conditions before the actual tests by walking once or twice with the metronome.

### 2.4. Data Analysis

The overground walking data were collected at 100 Hz and filtered using a second-order Butterworth low-pass filter with a 10-Hz cut-off frequency [15,16]. To collect consecutive steps under steady-state conditions, acceleration and deceleration steps were excluded (Figure 2a). Analyzed steps were as follows: slower: 29 ± 8 steps; preferred: 26 ± 6 steps; and faster: 24 ± 6 steps. 

Gait events were heel strikes and toe-offs that occurred when linear accelerations reached their maximum values on the anteroposterior and vertical axes, respectively, during a gait cycle [15] (Figure 2b).

Spatiotemporal parameters, including cadence, walking speed, stride length, and phases for single support, double support, and stance, were calculated. Gait asymmetry (GA) was calculated as differences between left and right movements during walking [21]. The percentage CV values ([standard deviation/mean] × 100) for all spatiotemporal parameters were calculated.

### 2.5. Statistical Analysis

All statistical analyses were performed using SPSS for Windows (version 21.0, SPSS Inc. Chicago, IL). The Shapiro-Wilk test was used to determine whether data were normally distributed. To verify slower and faster speed estimations, an ICC (2,1) analysis assessed the reliability of the estimated and measured speeds, as previously described by Noh et al. [19]. Estimated speed was defined as the calculated speed based on the measured preferred walking speed. If the measured preferred walking speed was 1.0 m/s, the estimated slower and faster speeds would be 0.8 and 1.2 m/s, respectively. The estimated speeds were compared with the measured speeds (actual speeds) during the slower and faster gait tasks. The limits of agreement (LOAs) were calculated according to the Bland–Altman plots to identify differences between the two systems [22].

An independent samples *t*-test was used to compare the differences between PD patients and controls. A one-way repeated measures analysis of variance with a Bonferroni correction (*p*-value: 0.05/3, 0.0167) was used to compare differences between slower, preferred, and faster speed conditions. Z-normalization (value-mean/standard deviation) was performed to normalize the tested variables. Stepwise binary logistic regression analysis was conducted to determine which variables were classifiers for PD patients based on gait-related variables. Pearson’s product-moment correlation analysis was used to determine the relationships between clinical data and gait variables. Statistical significance was set at 0.05.

## 3. Results

### 3.1. Reliability of Estimation and Measurement Speeds 

**Figure 3:**
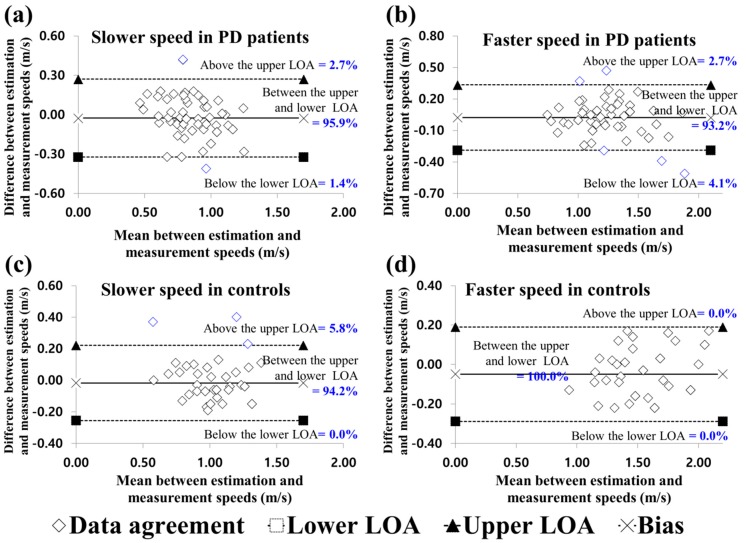
Bland-Altman plots for data agreement between the estimated and measured overground walking speeds. (**a**) and (**b**) are the slower and faster speed results for PD patients; (**c**) and (**d**) are the slower and faster speed results for control patients. PD, Parkinson’s disease; LOA, limits of agreement.

Table 2 shows the reliability of slower and faster walking speeds, indicating excellent agreement for PD patients and controls. Figure 3 shows that degrees of agreement at slower and faster speeds for PD patients and controls were 94.2–95.9% and 93.2–100.0%.

### 3.2. Group Differences: PD Patients vs. Controls

PD patients exhibited significantly lower walking speed and stride length than controls in all speed conditions. PD patients exhibited significantly higher GA (slower speed, *p* = 0.026; preferred speed, *p* = 0.045) and CV of stride length (slower speed, *p* = 0.015; preferred speed, *p* = 0.038) than controls at slower and preferred speed conditions. PD patients demonstrated a greater stance phase CV than controls at slower (*p* = 0.023) and faster (*p* = 0.045) speed conditions. Phases for single support (*p* = 0.034) and double support (*p* = 0.014), and CVs of single support (*p* = 0.033) and double support (*p* = 0.014) were significantly different between PD patients and controls, but only in the faster speed condition (Table 3).

### 3.3. Speed Differences: Slower, Preferred, and Faster Speed Conditions

All spatiotemporal parameters such as walking speed, stride length, and phases for single support, double support, and stance were significantly different among slower, preferred, and faster speed conditions for both PD patients and controls. PD patients demonstrated significantly decreasing trends for CVs of stride length (slower vs. preferred) and phases for single support (slower vs. preferred; slower vs. faster), double support (slower vs. preferred; slower vs. faster), and stance (slower vs. preferred). Stance phase CV within preferred and faster speed conditions showed a significantly increasing trend in PD patients. Controls exhibited significantly decreasing trends for CVs of stride length (slower vs. preferred; slower vs. faster) and phases for single support (all), double support (slower vs. faster), and stance (all) (Table 3).

### 3.4. Classifier Variables for PD Patients and Controls

Stepwise binary logistic regression analysis for the PD patients and controls revealed that the stride length was significantly different at the slower (odds ratio [OR]: 0.349, 95% confidence interval [CI]: 0.215–0.567, Nagelkerke R^2^ [R_N_^2^] = 0.233, *p* < 0.001) and preferred (OR: 0.274, 95% CI: 0.161–0.470, R_N_^2^ = 0.308, *p* < 0.001) speeds. At faster speeds, the walking speed and CV of the double support phase (walking speed OR: 0.231, 95% CI: 0.133–0.404, *p* < 0.001; CV of the double support phase OR: 2.083, 95% CI: 1.255–3.456; R_N_^2^ = 0.420, *p* = 0.005) were significantly different.

### 3.5. Relationship between Clinical Data and Gait Variables of PD Patients

At slower speeds, the UPDRS total was positively correlated with the stance phase (r = 0.251, *p* < 0.05), GA (r = 0.246, *p* < 0.05), CVs of stride length (r = 0.263, *p* < 0.05), the single support phase (r = 0.232, *p* < 0.05), and the stance phase (r = 0.275, *p* < 0.05). The UPDRS part 3 was positively correlated with GA (r = 0.333, *p* < 0.01), and the CV of the single support phase (r = 0.237, *p* < 0.05) and stance phase (r = 0.299, *p* < 0.05). At preferred speeds, the UPDRS total was positively correlated with the double support phase (r = 0.262, *p* < 0.05) and stance phase (r = 0.250, *p* < 0.05), and was negatively correlated with the single support phase (r = −0.229, *p* < 0.05). At faster speeds, the UPDRS part 3 was positively correlated with the double support phase CV (r = 0.299, *p* < 0.01) (Table 4).

## 4. Discussion

The main findings of this study are as follows: (1) The reliability of slower and faster estimated and measured speeds indicated excellent agreement between PD patients and controls. (2) PD patients demonstrated higher GA and CVs of stride length and stance phase at slower speeds, and higher CVs of phases for single support, double support, and stance than controls. (3) The double support phase CV was an important variable for distinguishing PD patients from controls at faster speeds. (4) Increased GA and CVs of stride length and phase-related variables at slower and faster speeds were associated with severe motor symptoms, indicating an increased UPDRS total and UPDRS part 3 score in PD patients.

Our PD patients exhibited a slower walking speed and shorter stride length than controls at all speed conditions, which is similar to previous findings [1,2,3,4,6,20]. In addition, PD patients demonstrated higher CVs for spatiotemporal parameters at slower and faster speeds, and higher GA at slower and preferred speeds than controls. Compared to the preferred speed, slower and faster speeds require more mechanical energy, which may influence the efficiency of energy use and alterations in muscle activity [6,23]. These mechanisms may contribute to increases in gait variability and GA in PD patients. Slower walking speeds led to increased time spent in the single-limb stance, and increased mediolateral displacement of the center of mass [6,24], thereby requiring more attention and dynamic stability [7]. Maintaining gait symmetry in this situation may be difficult even for healthy participants [24]; thus, our PD patients may exhibit higher values for gait variability and GA.

The double support phase CV shown at faster speeds was 2.1 times greater in the PD patients compared to controls, indicating that faster walking was more challenging for PD patients. Gait disturbances or even the freezing of gait (FOG) in PD patients may result from other similarly challenging tasks such as dual tasks or turning affect gait ability [4,20,25]. These gait disturbances may be related to basal ganglia dysfunction, which causes impairments in voluntary and involuntary movements [26,27]. Further, substantia nigra degeneration reflects a loss of dopaminergic innervation and posterior putamen dysfunction, which may influence the control of automatized behavior; therefore, PD patients may experience difficulty walking in an automatic manner without attention [20]. These abnormal motor functions may also result in impaired balance control [28], because PD patients may experience difficulties making an appropriate lateral weight shift onto the stance leg preceding a step to unload the stepping leg [20]. These symptoms may contribute to the increased double support phase in PD patients [6,28].

PD patients exhibit altered gait patterns during complex gait tasks such as turning in a dual task, which may be related to the increased risk of injuries and falls [25]. Complex gait tasks require additional cognitive loads for movement planning and processing of internal or external stimuli, which leads to increased volitional control, reduced automaticity, and increased gait instability [25,26]. These factors contribute to fragmented gait patterns when PD patients require a higher-level of cognitive control for gait [25]. We instructed participants to approximate the indicated slower and faster walking speeds as closely as possible. These conditions may have necessitated additional cognitive resources in PD patients compared to that for preferred speeds, as reported previously [25]. Gait tasks in the faster speed condition may be more challenging compared to tasks performed at the preferred speed even during the ON-state, which enhances the motor control of walking in PD patients [17]. Except for dysfunction of the basal ganglia, one contributor to gait disturbances is locomotor network dysfunction, such as cortical regions in PD patients [20]. Therefore, gait analysis at faster speeds may be useful for distinguishing gait characteristics of PD patients and controls, and the double support phase CV may be a variable that distinguishes the two groups.

We observed that the UPDRS total score was positively correlated with phases for double support and stance, and was negatively correlated with the single support phase at the preferred speed. Thus, PD patients with severe motor symptoms exhibit slower walking speeds [1,2,3,4,6], which may reflect the decline in gait ability. Furthermore, the UPDRS total score was positively related with stance phase, GV, and the CVs of stride length, the single support phase, and the stance phase at the slower speed. In addition, the UPDRS part 3 was associated with the GA and CV of the single support phase and stance phase at slower speeds, and with the double support phase CV at faster speeds. This optimization of walking in the self-preferred speed condition may be related to the interaction of neural and biomechanical mechanisms, which minimize active control of high-level sensory feedback control [29]. Slower or faster speed conditions are more challenging gait tasks than preferred speed conditions [12]. The slower speed condition requires a higher attention-demand, even in healthy young adults, because it results in reduced gait automaticity and higher cortical control with changes in muscle activity pattern [12,30,31]. PD patients may experience more difficulty with slower or faster speed conditions because they exhibit reduced gait automaticity, even under preferred speed conditions [20]; thus, these conditions may detect greater declines in gait ability [12]. The consideration of slower, faster, and preferred speed is vital when conducting advanced gait analysis and evaluating gait ability in clinical environments.

This study had several limitations. First, we did not consider the OFF-state condition in PD patients. Most PD patients live in the ON-state condition in their daily lives [17]; therefore, it is unclear how gait characteristics differ at slower and faster speeds between OFF- and ON-states. Second, we attempted to collect consecutive steps per trial (slower: 29 ± 8 steps; preferred: 26 ± 6 steps; faster: 24 ± 6 steps). Collecting more consecutive steps may increase the accuracy of the data. Thus, collecting as many steps as possible is recommended when performing gait analysis during overground walking tasks. Third, we were unable to include the scores obtained in the “New freezing of gait questionnaire” and the “Postural instability and gait disturbances” in our analysis, due to the small sample size of this study. Finally, we speculated that the slower or faster speed conditions might require a higher cognitive load in participants; however, our study did not measure the function of the cerebral cortex using functional magnetic resonance imaging.

## 5. Conclusions

We found that PD patients exhibited poorer gait stability at slower and faster speeds. Faster speeds may be useful for evaluating gait characteristics to distinguish PD patients and controls. Further, the double support phase CV was a classifier between the two groups. We therefore suggest that slower, faster, and preferred speeds during gait analysis and characteristics related to gait variability may be useful to evaluate the degree of motor symptoms in PD patients. Future studies should evaluate gait stability according to motor symptoms (faller and non-faller or with and without FOG) in PD patients at these speed conditions during the overground walking task.

## Figures and Tables

**Table 1:** Demographic characteristics of the study participants.

Variables	PD (*n* = 74)	Controls (*n* = 52)
Sex (male/female)	45/29	27/25
Age (years)	69.6 ± 5.7	71.4 ± 4.9
Height (cm)	159.8 ± 8.8	160.2 ± 9.4
Body weight (kg)	61.5 ± 8.7	64.6 ± 9.7
MMSE score	26.9 ± 2.4	27.0 ± 2.2
UPDRS total score	61.6 ± 18.4	-
UPDRS part 3 total score	40.9 ± 13.3	-
PIGD (score)	4.2 ± 3.2	-
Hoehn & Yahr scale	2.3 ± 0.3	-
L-dopa dose (mg/day)	788.7 ± 503.8	-
Non-Freezer (male/female)	15/5	-
NFOGQ (score)	14.8 ± 7.0	

Mean ± standard deviation. L-dopa: levodopa; MMSE: Mini-mental State Examination; NFOGQ: New freezing of gait questionnaire; PD: Parkinson’s disease; PIGD: Postural instability and gait disturbances; UPDRS: Unified Parkinson’s Disease Rating Scale.

**Table 2:** Reliability of the results for slower and faster walking speeds.

Variables	PD (*n* = 76)	Controls (*n* = 52)
Slower speed		
Estimated/measured (m/s)	0.86/0.89	1.03/1.05
ICC (2,1)	0.828	0.895
*p*-value	< 0.001	< 0.001
Faster speed		
Estimated/measured (m/s)	1.29/1.27	1.55/1.60
ICC (2,1)	0.905	0.945
*p*-value	< 0.001	< 0.001

ICC: Intraclass correlation of coefficient; PD: Parkinson’s disease.

**Table 3:** Group and speed differences of gait variables in PD patients and controls.

Variables	Slower Speed		Preferred Speed		Faster Speed		Significance for Groups	Significance for Speeds	
	PD Patients	Controls	PD Patients	Controls	PD Patients	Controls		PD Patients	Controls
Walking speed (m/s)	0.89 ± 0.21	1.05 ± 0.20	1.08 ± 0.21	1.29 ± 0.23	1.27 ± 0.28	1.60 ± 0.27	A, B, C	d, e, f	d, e, f
Stride length (m)	1.02 ± 0.22	1.20 ± 0.19	1.11 ± 0.22	1.33 ± 0.17	1.21 ± 0.24	1.46 ± 0.19	A, B, C	d, e, f	d, e, f
Single support phase (%)	38.72 ± 1.90	39.16 ± 1.66	39.87 ± 1.66	40.26 ± 1.63	41.06 ± 1.84	41.73 ± 1.80	C	d, e, f	d, e, f
Double support phase (%)	22.44 ± 3.84	21.56 ± 3.06	20.10 ± 3.26	19.41 ± 2.99	17.64 ± 3.46	16.37 ± 2.96	C	d, e, f	d, e, f
Stance phase (%)	61.16 ± 2.39	60.72 ± 1.70	59.97 ± 1.90	59.67 ± 1.58	58.70 ± 2.00	58.10 ± 1.45	N/S	d, e, f	d, e, f
GA (%)	3.79 ± 3.57	2.49 ± 2.58	2.88 ± 2.24	2.08 ± 2.12	2.78 ± 3.10	2.44 ± 2.35	A, B	N/S	N/S
CV of stride length (%)	3.05 ± 1.48	2.47 ± 1.00	2.31 ± 1.40	1.86 ± 0.81	2.51 ± 3.78	1.85 ± 0.76	A, B	d	d, e
CV of single support phase (%)	4.69 ± 3.14	4.34 ± 2.26	3.10 ± 1.70	2.65 ± 0.90	2.68 ± 1.50	2.19 ± 0.71	C	d, e	d, e, f
CV of double support phase (%)	11.38 ± 6.00	9.48 ± 4.25	9.10 ± 5.21	7.98 ± 3.68	9.40 ± 4.58	7.48 ± 3.75	C	d, e	e
CV of stance phase (%)	4.51 ± 2.40	3.62 ± 1.69	2.91 ± 1.84	2.48 ± 1.28	2.67 ± 1.74	1.99 ± 0.85	A, C	d, e	d, e, f

CV: coefficient of variance, GA: gait asymmetry, PD: Parkinson’s disease. Group differences between PD and controls for slower (A), preferred (B), and faster (C) speeds, *p* < 0.05 Speed differences within Slower vs. Preferred (d), Slower vs. Faster (e), Preferred vs. Faster (f), *p* < 0.0167 (0.05/3), N/S indicates no significance.

**Table 4:** Pearson’s product moment correlation analysis between clinical data and gait variables of PD patients at slower, preferred, and faster speeds.

Variables	UPDRS Total	UPDRS Part 3	L-Dopa Dose
Slower Speed			
Stance phase	0.251 *	0.185	0.010
GA	0.246 *	0.333 **	−0.174
CV of stride length	0.263 *	0.219	0.016
CV of single support phase	0.232 *	0.237 *	−0.039
CV of stance phase	0.275 *	0.299 *	−0.074
Preferred Speed			
Single support phase	−0.229 *	−0.175	0.031
Double support phase	0.262 *	0.161	0.022
Stance phase	0.250 *	0.123	0.064
Faster Speed			
CV of double support phase	0.202	0.299 **	−0.179

CV: coefficient of variance, GA: gait asymmetry, L-dopa: levodopa, UPDRS: Unified Parkinson’s Disease Rating Scale. * *p* <0.05, ** *p* < 0.01.

## Supplementary Materials

The following are available online at https://www.mdpi.com/1424-8220/20/7/2132/s1, Supplementary file S1: IRB number; Supplementary file S2: Research data (raw data); Supplementary Table S1: List of levodopa-equivalent dose.
